# Contribution of prepregnancy body mass index and gestational weight gain to adverse neonatal outcomes: population attributable fractions for Canada

**DOI:** 10.1186/s12884-015-0452-0

**Published:** 2015-02-05

**Authors:** Susie Dzakpasu, John Fahey, Russell S Kirby, Suzanne C Tough, Beverley Chalmers, Maureen I Heaman, Sharon Bartholomew, Anne Biringer, Elizabeth K Darling, Lily S Lee, Sarah D McDonald

**Affiliations:** Maternal and Infant Health Section, Health Surveillance and Epidemiology Division, Public Health Agency of Canada, 785 Carling Avenue, 6804A 4th Floor, Ottawa, Ontario K1A 0 K9 Canada; Reproductive Care Program of Nova Scotia, Halifax, Nova Scotia Canada; Department of Community and Family Health, College of Public Health, University of South Florida, Tampa, FL U.S.A; Departments of Paediatrics and Community Health Sciences, Faculty of Medicine, University of Calgary, Calgary, Alberta Canada; Department of Obstetrics and Gynaecology, Ottawa Hospital Research Institute, University of Ottawa, Ottawa, Ontario Canada; College of Nursing, Faculty of Health Sciences, University of Manitoba, Winnipeg, Manitoba Canada; Department of Family and Community Medicine, University of Toronto, Mount Sinai Hospital, Toronto, Ontario Canada; Midwifery Education Program, Laurentian University, Sudbury, Ontario Canada; Perinatal Services British Columbia, Provincial Health Services Authority, Vancouver, British Columbia Canada; Departments of Obstetrics & Gynecology, Radiology, and Clinical Epidemiology & Biostatistics, McMaster University, Hamilton, Canada

**Keywords:** Population attributable fraction, Maternal weight, Preterm birth, Small-for-gestational age, Large-for-gestational age

## Abstract

**Background:**

Low or high prepregnancy body mass index (BMI) and inadequate or excess gestational weight gain (GWG) are associated with adverse neonatal outcomes. This study estimates the contribution of these risk factors to preterm births (PTBs), small-for-gestational age (SGA) and large-for-gestational age (LGA) births in Canada compared to the contribution of prenatal smoking, a recognized perinatal risk factor.

**Methods:**

We analyzed data from the Canadian Maternity Experiences Survey. A sample of 5,930 women who had a singleton live birth in 2005-2006 was weighted to a nationally representative population of 71,200 women. From adjusted odds ratios, we calculated population attributable fractions to estimate the contribution of BMI, GWG and prenatal smoking to PTB, SGA and LGA infants overall and across four obstetric groups.

**Results:**

Overall, 6% of women were underweight (<18.5 kg/m^2^) and 34.4% were overweight or obese (≥25.0 kg/m^2^). More than half (59.4%) gained above the recommended weight for their BMI, 18.6% gained less than the recommended weight and 10.4% smoked prenatally. Excess GWG contributed more to adverse outcomes than BMI, contributing to 18.2% of PTB and 15.9% of LGA. Although the distribution of BMI and GWG was similar across obstetric groups, their impact was greater among primigravid women and multigravid women without a previous PTB or pregnancy loss. The contributions of BMI and GWG to PTB and SGA exceeded that of prenatal smoking.

**Conclusions:**

Maternal weight, and GWG in particular, contributes significantly to the occurrence of adverse neonatal outcomes in Canada. Indeed, this contribution exceeds that of prenatal smoking for PTB and SGA, highlighting its public health importance.

## Background

Low or high prepregnancy body mass index (BMI) and inadequate or excess gestational weight gain (GWG) are linked to an increased risk of adverse neonatal outcomes. Overweight and obese BMI and excess GWG have been associated with large-for-gestational age (LGA) infants, preterm birth (PTB) and stillbirth while underweight BMI and low GWG have been associated with small-for-gestational age (SGA) infants and PTB [1-6]. Although elevated individual-level risks have been documented in numerous studies, less is known about population-level effects of both BMI and GWG on neonatal outcomes.

Population-level effects can be measured using population attributable fractions (PAFs), which reflect the increased risk conferred by a particular determinant and its prevalence in the population. The PAF provides a hypothetical assessment of the proportion of the outcome that could be avoided if a particular risk factor were eliminated, making it an important outcome measure from a public health perspective. A 2011 systematic review and meta-analysis of risk factors for stillbirth in high-income countries concluded that 8-18% of stillbirths were attributable to maternal overweight and obesity (BMI ≥ 25 kg/m^2^) [1].

In Canada, based on measured height and weight, the prevalence of obesity among adult women rose from 16% in 1978 to 23% in 2004 [7], reflecting a global trend in increasing overweight and obesity [8]. If this trend continues, as hypothesized by Cnattinguis et al. [9], the public health importance of maternal overweight and obesity may rival prenatal smoking as a modifiable risk factor for adverse pregnancy outcomes. The objective of this study is to examine this hypothesis for Canada, by estimating the contribution of BMI and GWG, as measured by PAFs, to PTB, SGA and LGA and to compare this to the contribution of prenatal smoking, a recognized perinatal risk factor.

## Methods

### Study population

We used data from the Public Health Agency of Canada’s Canadian Maternity Experiences Survey (MES). The MES was a cross-sectional survey of a stratified random sample of women drawn from the 2006 Canadian Census. Women who had a singleton live birth in Canada between November 2005 and May 2006, were at least 15 years of age, living with their infant, and not living on a First Nations reserve or in an institution were eligible to participate. Of 8,244 eligible women, 6,421 (78%) agreed to participate. Data were collected by female interviewers between October 2006 and January 2007 using a computer-assisted telephone interview application. Women had previously been mailed a letter which included information on the survey and asked for their participation. Verbal consent to participate was then obtained at the beginning of each telephone interview. All data in this study were based on women’s reports during survey interviews.

For this study, we excluded 491 women who were either missing information on BMI or GWG (the principal determinants of interest), missing information on gestational age (needed for outcome variables) or were less than 18 years old because the BMI classification we used was derived for ages 18 and older. In consideration of the sample design and non-response, each MES participant was assigned a sampling weight. The 5,930 women in this study were thus weighted to a nationally representative population of 71,200 women who had a singleton live birth in Canada between November 2005 and May 2006. Sampling weights took into consideration the probability of each respondent being selected from the stratified sample frame and also adjusted for non-response. Using Census data, comparison of the respondent distribution using the weights to the target population showed a close approximation on all demographic characteristics investigated (e.g., maternal age and household composition). Detailed information on the survey’s development, including the weighting procedure and analysis of respondent characteristics has been reported elsewhere [10,11].

### Outcomes

We studied three outcomes: PTB (<37 weeks completed gestation), SGA (weight below the 10^th^ percentile for gestational age) and LGA (weight above the 90^th^ percentile for gestational age). Infants were classified as SGA or LGA using MES data on mother’s country of birth and sex-specific growth curves developed by Ray et al. [12]. Ray et al.’s growth curves are specific to mother’s country of birth grouped according to seven world regions (Canada, Europe/Western nations, Africa/Caribbean, Middle East/North Africa, Latin America, East Asia/South East Asia/Pacific Islands, South Asia). Accounting for mother’s country of birth minimizes the risk of misclassifying newborns of mothers from non-European backgrounds as too small or too large for their gestational age due to recognized non-pathological ethnic differences in birthweight [12,13]. Ray et al.’s growth curves end at 41 weeks gestation and our sample included 90 infants with gestational ages ranging from 42 to 45 weeks. These infants were classified using the 41-week 10^th^ and 90^th^ percentile cut-offs. Fetal growth after 41 weeks gestation could have resulted in a baby being classified in a different growth category (e.g. AGA) than he/she would have been at 41 weeks (e.g., SGA); however any minor misclassification which may have resulted from this approach was deemed preferable to eliminating these records.

### Determinants

Prepregnancy BMI and GWG measures were derived from the following questions:

i) *How tall are you without shoes on?*
ii) *Just before your pregnancy, how much did you weigh?*
iii) *How much weight did you gain during your pregnancy?*

We categorized women’s prepregnancy weight according to the World Health Organization (WHO) standard as either being underweight (BMI < 18.5 kg/m^2^), normal weight (18.5 ≤ BMI < 25), overweight (25 ≤ BMI < 30) or obese (BMI ≥ 30).

We utilized the 2009 Institute of Medicine (IOM) guidelines on GWG to categorize women’s weight gain as below, within or above recommended [14]. These guidelines have also been adopted by Health Canada [15]. Because GWG is associated with gestational length, we accounted for gestational length in our calculations. Specifically, we assumed a 2 kg weight gain in the first trimester (as per IOM guidelines) and subtracted this amount from the total reported weight gain to obtain GWG during the remainder of the pregnancy. Next, we subtracted 13 weeks (the first trimester) from the gestational age at birth to obtain the number of weeks in the remainder of the pregnancy. We then compared the GWG in the remainder of the pregnancy to the IOM’s recommended GWG during this period, accounting for women’s prepregnancy BMI.

Prenatal smoking was determined from the question “During the last 3 months of your pregnancy, did you smoke daily, occasionally, or not at all?” Women who responded either daily or occasionally were categorized as smokers.

### Covariates

We studied additional reproductive, health care, sociodemographic and psychosocial characteristics as potential confounders of the association between BMI, GWG and neonatal outcomes. Our selection of covariates was guided by previous studies of risk factors for PTB, SGA and LGA [3,4,6]. Table 1 provides definitions of the variables that are not self-explanatory.

**Table 1:** **Definition of covariate variables**

**Variable**	**Definition**
Low-income-cut-off (LICO) [16]	A measure of the income threshold below which a family will likely spend 20 percentage points more than the average family on food, shelter and clothing
Medical condition prior to pregnancy	Yes in response to the question “Prior to your pregnancy, did you develop any medical conditions or health problems that required you to take medication for more than 2 weeks, have special care, or extra tests during pregnancy?”
Medical condition during pregnancy	Yes in response to the question “During your pregnancy, did you develop any new medical conditions or health problems that required you to take medication for more than 2 weeks, have special care, or extra tests?”
Depression prior to pregnancy	Yes in response to the question “Before your pregnancy, had you ever been prescribed anti-depressants or been diagnosed with depression?
Short stature	Height of less than 1.55 metres (5 feet 2 inches)

### Statistical analysis

Percentages were used to report observed distributions of BMI, GWG, prenatal smoking and other maternal characteristics. Using multivariable logistic regression, we calculated adjusted odds ratios (aORs) separately for each outcome (PTB, SGA and LGA). For SGA analyses, the sample was restricted to infants who were SGA and infants who were average-for-gestational-age (AGA). Similarly, LGA analyses were restricted to infants who were LGA and AGA. BMI, GWG and smoking were included in all multivariable models in order to estimate their independent associations with each outcome. Other covariates were selected purposely into models using the following steps. Based on the Wald test from univariable logistic regression models, we initially included any variable with a p-value below 0.25 [17]. Covariates were then removed from the model if they were statistically non-significant and not a confounder. Significance was evaluated at the 0.05 level and confounding as a change of 15% or greater in the effect of BMI, GWG or smoking on the outcome being modeled. No adjustment for multiple comparisons was used.

With the exception of maternal age, all variables were treated as categorical in regression models. Records with missing values for covariates other than LICO were excluded from models (<4%). Due to a large number of missing LICO values (8.0%), a missing category was included for this variable. Calculations were carried out overall, and for four mutually exclusive obstetric history groups: primigravidas, multigravidas with a previous PTB, multigravidas without a previous PTB but with a previous early pregnancy loss (miscarriage, abortion, and/or ectopic pregnancy) and multigravidas without a previous PTB or previous early pregnancy loss. Analysis by these groups was based on prior knowledge that pregnancy outcomes differ by parity and for women with antecedent adverse outcomes such as PTB [18,19]. Normal BMI, within recommended GWG and non-smokers were the reference groups.

The contribution of maternal underweight, overweight or obese BMI, less than or more than recommended GWG, and smoking to each outcome was estimated using PAFs. We calculated adjusted PAFs using the sequential and average attributable fraction method [20]. This method involves estimating a logistic regression model with known/available confounders for each outcome, then using this model to compute the adjusted number of cases that would be expected if the risk factor (e.g., excess GWG) were absent in the population. The adjusted PAF is calculated by subtracting this number of expected cases from the number of observed cases, then dividing this value by the number of observed cases.

All analyses were carried out using sampling weights. Results were computed from unrounded weighted components; however weighted sample sizes in results tables were rounded to the nearest hundred, as unrounded estimates overstate precision. We calculated 95% confidence intervals for adjusted ORs and adjusted PAFs using the bootstrap method, which accounts for the variability introduced by the sample design and weighting adjustments [21]. Bootstrap confidence intervals were based on 1,000 samples. We used SAS® Enterprise Guide® software, Version 5.1 [22].

The MES project was presented to Health Canada’s Science Advisory Board, Health Canada’s Research Ethics Board and the Federal Privacy Commissioner, and was approved by Statistics Canada’s Policy Committee [11]. Additional ethical review was not required, as the MES data are anonymous and this study did not generate identifying information.

## Results

The distribution of BMI, GWG, smoking and covariates is presented in Table 2. Overall, 6% of women were underweight and 34.4% were either overweight or obese. More than half (59.4%) gained above the recommended weight for their BMI; 18.6% gained less than recommended weight; and smoking was reported by 10.4% of women. The distribution of maternal characteristics varied across obstetric history groups, with women with a previous PTB reporting the highest rates of obesity (19.6%) and prenatal smoking (17.2%) and primigravidas reporting the highest rates of above recommended GWG (61.3%). Among women with above recommended GWG, the average weight gain among primigravidas was also higher (20.4 kg) than that among multigravidas (18.9 kg). Across other characteristics, women with a previous PTB tended to differ from other groups of women. These differences included lower educational achievement, having a household income that was at or below the LICO, having medical conditions prior to pregnancy or during pregnancy, being diagnosed with depression prior to pregnancy and experiencing higher levels of stress and lower social support during their pregnancy. Among the women who reported an early pregnancy loss, 68.2%, 36.4% and 2.7% experienced a miscarriage, abortion and ectopic pregnancy, respectively.

**Table 2:** Percent of population with maternal characteristics*

	**All women (n = 71,200)**	**Primigravidas (n = 24,000)**	**Multigravidas**		
			**Previous PTB (n = 4,000)**	**No previous PTB**	
				**Previous early pregnancy loss** ^**a**^ **(n = 21,300)**	**No previous early pregnancy loss (n = 21,600)**
Percent of sample		33.8	5.7	30.0	30.5
*Determinants*					
Prepregnancy BMI					
<18.5	6.0	7.1	6.2	5.3	5.4
18.5-24.9	59.7	63.3	51.3	58.1	58.4
25.0-29.9	20.9	17.6	22.9	23.1	22.3
≥30	13.5	12.0	19.6	13.5	13.9
Gestational weight gain					
<recommended	18.6	16.6	18.0	19.4	20.2
=recommended	23.0	22.1	23.2	22.3	24.9
>recommended	59.4	61.3	58.7	58.3	54.9
Smoked 3^rd^ trimester	10.4	7.7	17.2	13.7	10.0
*Covariates*					
Maternal age					
≤24	15.3	23.6	11.9	12.4	9.5
25-29	34.1	40.4	27.7	29.0	33.5
30-34	33.4	26.1	37.7	35.4	37.8
≥35	17.5	9.8	22.7	23.2	19.3
Maternal education					
Less than high school	6.8	5.1	12.4	7.8	6.7
High school graduate	19.4	17.7	22.7	20.3	19.8
Post-secondary diploma	37.7	38.1	41.6	36.8	37.4
University graduate	36.1	39.2	23.3	35.2	36.1
Low-income-cut-off (LICO)					
≤LICO	18.0	14.9	26.2	19.1	19.1
>LICO	74.0	75.3	69.1	74.1	74.0
Missing	8.0	9.8	4.7	6.9	6.9
Short stature	6.4	5.6	6.8	5.8	7.7
World region of birth					
Canada	76.7	77.8	82.2	76.4	74.9
Europe/Western nations	6.0	5.8	4.4	6.4	5.9
Africa/Caribbean	2.3	2.2	3.4	2.6	1.9
Middle East/North Africa	2.6	2.6	2.3	1.9	3.3
Latin America	2.3	1.8	1.3	2.2	3.2
East Asia/South East Asia/Pacific Islands	5.7	5.4	3.0	6.4	5.7
South Asia	4.5	4.5	3.5	4.1	5.1
Medical conditions prior to pregnancy	15.2	14.3	20.5	16.6	13.8
Medical conditions during pregnancy	24.4	24.8	32.2	25.1	21.8
Depression prior to pregnancy	15.5	13.8	20.2	17.3	14.6
Perceived stress during pregnancy					
Not stressful	43.1	46.4	34.2	41.6	42.6
Somewhat stressful	44.9	44.7	47.6	44.7	45.0
Very stressful	12.0	8.9	18.2	13.8	12.4
No support during pregnancy	12.8	10.0	16.8	14.7	13.3

*Subgroup sample sizes do not add up to the total due to rounding to the nearest 100, as explained in the methods. Abbreviations: PTB – preterm birth, BMI – body mass index.

^a^Early pregnancy loss - miscarriage, abortion and/or ectopic pregnancy.

### Associations between adverse neonatal outcomes and BMI and GWG

Table 3 presents associations between adverse neonatal outcomes and BMI and GWG.

**Table 3:** Rate (%) and adjusted odds ratios (aORs) of adverse neonatal outcomes associated with BMI, GWG and prenatal smoking*

	**PTB**		**SGA**		**LGA**	
	**%**	**aOR (95% CI)**	**%**	**aOR (95% CI)**	**%**	**aOR (95% CI)**
*All women* (n = 71,200)	6.1		8.1		11.3	
Prepregnancy BMI						
<18.5	8.4	1.55 [0.96 , 2.52]	20.5	**2.04 [1.46, 2.84]**	4.3	0.64 [0.33, 1.20]
18.5-24.9	5.6	Reference	10.0	Reference	7.5	Reference
25.0-29.9	6.1	0.98 [0.72, 1.33]	8.2	0.95 [0.74, 1.22]	11.4	**1.38 [1.08, 1.75]**
≥30	7.4	1.02 [0.73,1.42]	8.6	0.86 [0.63, 1.15]	14.1	**1.89 [1.45, 2.47]**
Gestational weight gain						
< recommended	6.5	1.35 [0.92, 1.97]	16.1	**1.56 [1.20, 2.03]**	4.6	0.57 [0.39, 0.84]
= recommended	4.5	Reference	10.9	Reference	7.7	Reference
> recommended	6.7	**1.45 [1.06, 1.98]**	7.7	0.70 [0.56, 0.87]	11.0	**1.34 [1.04, 1.72]**
Smoked 3^rd^ trimester						
Yes	8.9	1.31 [0.92, 1.86]	18.4	**2.06 [1.59, 2.67]**	2.8	0.26 [0.16, 0.43]
No	5.8	Reference	9.1	Reference	9.7	Reference
*Primigravidas* (n = 24,000)	6.8		10.5		7.5	
Prepregnancy BMI						
<18.5	8.7	1.63 [0.77, 3.47]	28.3	**2.47 [1.53, 3.99]**	0.8	0.14 [0.005, 4.69]
81.5-24.9	6.1	Reference	12.2	Reference	6.1	Reference
25.0-29.9	8.6	1.30 [0.80, 2.12]	10.4	0.89 [0.59, 1.34]	7.5	1.12 [0.67, 1.87]
≥30	6.6	0.86 [0.45, 1.66]	12.1	0.97 [0.61, 1.57]	11.4	**1.89 [1.11, 3.21]**
Gestational weight gain						
< recommended	6.6	1.60 [0.78, 3.29]	20.8	1.40 [0.92, 2.12]	2.1	0.49 [0.17, 1.41]
= recommended	3.9	Reference	15.9	Reference	4.1	Reference
> recommended	7.9	**2.02 [1.13, 3.63]**	9.9	0.61 [0.44, 0.86]	8.8	**2.01 [1.12, 3.61]**
Smoked 3^rd^ trimester						
Yes	8.2	1.04 [0.53, 2.04]	27.4	**2.32 [1.49, 3.62]**	2.1	0.29 [0.08, 1.13]
No	6.7	Reference	11.8	Reference	7.0	Reference
*Multigravidas, previous PTB* (n = 4,000)	13.3		7.4		10.9	
Prepregnancy BMI						
<18.5	8.6	0.70 [0.05, 9.26]	19.7	1.79 [0.20, 15.69]	0	--^b^
18.5-24.9	15.0	Reference	9.3	Reference	7.2	Reference
25.0-29.9	9.5	0.56 [0.19, 1.67]	6.8	1.14 [0.26, 4.92]	10.6	1.58 [0.33, 7.49]
≥30	14.9	0.89 [0.36, 2.22]	5.9	0.42 [0.08, 2.18]	3.8	0.55 [0.07, 4.34]
Gestational weight gain						
< recommended	13.1	0.83 [0.22, 3.10]	17.1	1.40 [0.35, 5.58]	6.9	1.24 [0.08, 18.45]
= recommended	15.6	Reference	12.8	Reference	4.6	Reference
> recommended	12.5	0.93 [0.33, 2.59]	4.5	0.30 [0.08, 1.14]	7.7	1.01 [0.14, 7.45]
Smoked 3^rd^ trimester						
Yes	16.0	1.45 [0.46, 4.61]	12.8	1.77 [0.43, 7.24]	0	--^b^
No	12.8	Reference	7.8	Reference	8.3	Reference
*Multigravidas, no previous PTB, previous loss* ^a^ (n = 21,300)	6.2		7.1		11.8	
Prepregnancy BMI						
<18.5	10.3	1.90 [0.73, 4.93]	14.2	1.75 [0.84, 3.66]	6.7	1.04 [0.37, 2.90]
18.5-24.9	5.7	Reference	9.2	Reference	7.5	Reference
25.0-29.9	5.9	1.00 [0.57, 1.75]	6.4	0.72 [0.44, 1.19]	12.3	1.53 [1.00, 2.36]
≥30	7.2	0.92 [0.47, 1.80]	8.3	0.84 [0.46, 1.54]	16.9	**2.61 [1.61, 4.23]**
Gestational weight gain						
< recommended	8.1	1.14 [0.59, 2.19]	14.2	1.65 [0.96, 2.83]	5.0	0.41 [0.20, 0.81]
= recommended	5.9	Reference	8.2	Reference	9.9	Reference
> recommended	5.7	0.93 [0.53, 1.62]	7.1	0.83 [0.50, 1.36]	11.4	1.02 [0.64, 1.63]
Smoked 3^rd^ trimester						
Yes	8.8	1.77 [0.97, 3.23]	16.3	**2.26 [1.40, 3.64]**	10.8	0.23 [0.10, 0.51]
No	5.8	Reference	7.5	Reference	3.6	Reference
*Multigravidas, no previous PTB, no previous loss* ^a^ (n = 21,600)	4.0		6.7		14.9	
Prepregnancy BMI						
<18.5	6.1	2.14 [0.56, 8.20]	15.3	1.81 [0.84, 3.88]	6.9	0.93 [0.31, 2.76]
18.5-24.9	3.5	Reference	8.1	Reference	9.3	Reference
25.0-29.9	3.3	0.75 [0.37, 1.51]	8.3	1.24 [0.76, 2.03]	14.2	1.46 [0.99, 2.14]
≥30	6.5	1.21 [0.59, 2.50]	6.2	0.78 [0.38, 1.61]	16.8	**1.79 [1.14, 2.80]**
Gestational weight gain						
< recommended	3.8	1.98 [0.71, 5.51]	13.6	**1.76 [1.03, 3.03]**	6.2	0.66 [0.34, 1.26]
= recommended	2.1	Reference	8.2	Reference	9.9	Reference
> recommended	4.9	2.52 [0.99, 6.37]	6.4	0.80 [0.49, 1.30]	13.8	1.36 [0.88, 2.08]
Smoked 3^rd^ trimester						
Yes	7.0	1.13 [0.50, 2.59]	15.2	**1.92 [1.10, 3.35]**	3.3	0.29 [0.12, 0.71]
No	3.7	Reference	7.6	Reference	12.1	Reference

*Subgroup sample sizes do not add up to the total due to rounding to the nearest 100, as explained in the methods. Statistically significant aORs are bolded.

Abbreviations: PTB – preterm birth, SGA – small-for-gestational-age, LGA – large-for-gestational-age, BMI – body mass index.

^a^Early pregnancy loss – miscarriage, abortion and/or ectopic pregnancy.

^b^No cases of LGA in these subgroup.

#### Preterm birth

The PTB rate among all women was 6.1%, ranging from 4.0% among women with no previous PTB or pregnancy loss to 13.3% among women with a previous preterm birth. Observed rates were highest among women who were underweight or obese, women below or above their recommended GWG and among women who smoked. However, after adjusting for other maternal characteristics, only having above recommended GWG was significantly associated with preterm birth (overall and among primigravidas).

#### Small-for-gestational-age

The SGA birth rate was 8.1%, ranging from 6.7% among women with no previous PTB or pregnancy loss to 10.5% among primigravidas. Women who were underweight, were below their recommended GWG and smoked prenatally were significantly more likely to have an SGA baby.

#### Large-for-gestational-age

The LGA birth rate was 11.3%, ranging from 7.5% among primigravidas to 14.9% among multigravid women with no previous PTB or pregnancy loss. Women who were overweight or obese and were above their recommended GWG were significantly more likely to have an LGA baby. Women whose GWG was below recommendations and women who smoked prenatally were less likely to have an LGA baby.

Generally, the direction of effects for PTB, SGA and LGA remained the same across the four obstetric groups though the magnitude of effects and statistical significance of associations varied. BMI, GWG and smoking were more commonly associated with infant weight (either SGA or LGA) than with PTB. Of the three determinants, only GWG was significantly associated with all outcomes. The small number of women with a previous PTB limited the statistical power to detect significant associations in this group (Table 3).

### PAFs for adverse neonatal outcomes

Table 4 presents positive and negative PAFs for adverse neonatal outcomes. Negative PAFs reflect protective ORs in Table 3. We focus on positive PAFs, which estimate the independent contribution of each determinant to PTB, SGA and LGA (i.e., after adjusting for the other two determinants and other covariates).

**Table 4:** Adjusted population attributable fractions (PAFs) of adverse neonatal outcomes due to BMI, GWG and prenatal smoking*

		**PAF (%, 95 CI)**		
**Characteristic**	**Prevalence (%) of characteristic**	**PTB**	**SGA**	**LGA**
*All women* (n = 71,200)				
Underweight	6.0	2.6 [2.5, 2.7]	5.3 [5.2, 5.4]	−1.4 [-1.4, -1.3]
Overweight	20.9	−0.4 [-0.6, -0.2]	−0.8 [-0.9, -0.7]	6.5 [6.3, 6.6]
Obese	13.5	0.3 [0.1, 0.4]	−1.6 [-1.7, -1.5]	8.9 [8.8, 9.1]
< recommended GWG	18.6	4.7 [4.4, 5.0]	9.2 [9.0, 9.4]	−6.3 [-6.4, -6.2]
> recommended GWG	59.4	18.2 [17.8, 18.7]	−16.3 [-16.4, -16.1]	15.9 [15.4, 16.3]
Smoked 3^rd^ trimester	10.4	3.2 [3.0, 3.3]	8.7 [8.6, 8.8]	−7.1 [-7.2, -7.0]
*Primigravidas* (n = 24,000)				
Underweight	7.1	3.3 [3.1, 3.5]	7.5 [7.4, 7.7]	−4.6 [-4.7, -4.5]
Overweight	17.6	4.8 [4.5, 5.1]	−1.4 [-1.6, -1.3]	2.0 [1.7, 2.2]
Obese	12.0	−1.7 [-2.0, -1.5]	−0.1 [-0.2, 0.1]	8.9 [8.6, 9.1]
< recommended GWG	16.6	5.7 [5.5, 6.0]	6.1 [5.8, 6.5]	−5.1 [-5.3, -4.9]
> recommended GWG	61.3	33.9 [33.1, 34.6]	−22.8 [-23.4, -22.3]	37.9 [37.1, 38.6]
Smoked 3^rd^ trimester	7.7	0.3 [0.1, 0.5]	7.5 [7.4, 7.6]	−5.1 [-5.2, -5.0]
*Multigravidas, previous PTB* (n = 4,000)				
Underweight	6.2	−1.4 [-1.6 ,-1.2]	4.6 [4.2, 5.1]	--^a^
Overweight	22.9	−10.5 [-11.1, -9.9]	1.9 [1.3, 2.5]	--^a^
Obese	19.6	−2.1 [-2.6, -1.7]	−14.0 [-14.9,-13.2]	--^a^
< recommended GWG	18.0	−3.0 [-3.6, -2.4]	8.0 [7.1, 8.9]	--^a^
> recommended GWG	58.7	−3.6 [-5.1, -2.1]	−54.8 [-57.1, -52.4]	--^a^
Smoked in 3^rd^ trimester	17.2	5.4 [4.9, 6.0]	9.7 [9.0, 10.4]	--^a^
*Multigravidas, no previous PTB, previous loss* ^*b*^ (n = 21,300)				
Underweight	5.3	3.5 [3.3, 3.7]	3.2 [3.1, 3.4]	0.1 [0.0, 0.2]
Overweight	23.1	−0.05 [-0.4, 0.3]	−5.6 [-5.9, -5.4]	8.9 [8.6, 9.2]
Obese	13.5	−1.2 [-1.4, -0.9]	−2.2 [-2.4, -2.0]	13.0 [12.7, 13.2]
< recommended GWG	19.4	2.7 [2.3, 3.1]	11.0 [10.6, 11.4]	−11.4 [-11.6, -11.1]
> recommended GWG	58.3	−3.7 [-4.5, -2.8]	−8.6 [-9.3, -7.9]	1.1 [0.2, 1.9]
Smoked 3^rd^ trimester	13.7	8.0 [7.7, 8.2]	13.0 [12.7, 13.2]	−13.2 [-13.4,-13.0]
*Multigravidas, no previous PTB, no previous loss* ^*b*^ (n = 21,600)				
Underweight	5.4	4.2 [4.0, 4.5]	3.8 [3.7, 4.0]	−0.2 [-0.3, -0.1]
Overweight	22.3	−5.9 [-6.4, -5.5]	3.9 [3.6, 4.2]	7.6 [7.4, 7.8]
Obese	13.9	3.4 [3.0, 3.8]	−2.5 [-2.7, -2.3]	7.9 [7.7, 8.1]
< recommended GWG	20.2	9.5 [9.1, 9.9]	12.3 [11.6, 13.1]	−5.0 [-5.2, -4.8]
> recommended GWG	54.9	38.7 [37.8, 39.5]	−9.3 [-10.0, -8.6]	15.2 [14.6, 15.8]
Smoked 3^rd^ trimester	9.0	1.6 [1.2, 1.9]	7.0 [6.8, 7.2]	−5.4 [-5.4, -5.3]

*Subgroup sample sizes do not add up to the total due to rounding to the nearest 100, as explained in the methods.

Abbreviations: PAF – population attributable fraction, PTB – preterm birth, SGA – small-for-gestational-age, LGA- large-for-gestational-age, BMI – body mass index, GWG – gestational weight gain.

^a^PAFs cannot be estimated due to the small sample size / small number of LGA babies in this group.

^b^Early pregnancy loss – miscarriage, abortion and/or ectopic pregnancy.

#### Preterm birth

Among all women, above recommended GWG contributed to 18.2% of PTB, while low BMI, high BMI and prenatal smoking each contributed to less than 5% of PTB. The pattern among primigravidas and multigravidas with no previous PTB or pregnancy loss was similar to that among all women. Above recommended GWG contributed to over a third of PTB among primigravidas and multigravidas with no previous PTB or pregnancy loss (33.9% and 38.7% respectively). Among women who had experienced a previous PTB, only the PAF of prenatal smoking was positive (5.4%).

#### Small-for-gestational-age

Among all women, below recommended GWG contributed more (9.2%) to SGA births than prenatal smoking (8.7%) or underweight BMI (5.3%), although the odds of having an SGA baby were higher among women who smoked and were underweight (Table 3). Below recommended GWG, prenatal smoking and underweight BMI contributed to SGA births in all four obstetric groups. The highest PAF due to below recommended weight gain was among multigravidas with no previous PTB or pregnancy loss (12.3%); the highest PAF due to prenatal smoking was among multigravidas with no previous PTB but with a previous pregnancy loss (13.0%); and the highest PAF due to underweight BMI was among primigravidas (7.5%).

#### Large-for-gestational-age

Overall, above recommended GWG contributed more (15.9%) to LGA births than being overweight (6.5%) or obese (8.9%). Larger PAFs due to excess GWG were observed among primigravidas and multigravidas with no previous PTB or pregnancy loss, than among multigravidas with a previous pregnancy loss but no previous PTB. Similar to the results for PTB, above recommended GWG contributed to over a third (37.9%) of LGA births in primigravidas. It was not possible to estimate the PAFs for multigravidas with a previous PTB due to the small size of this group (Table 4).

## Discussion

The results of this nationally representative study indicate that low or high prepregnancy BMI and inadequate or excess GWG are important contributors to PTB, SGA and LGA infants in Canada. Due to almost 60% of women gaining above the recommended weight for their prepregnancy BMI, excess GWG contributed more to adverse outcomes than high BMI, to 18.2% of PTB and 15.9% of LGA overall. Although the distribution of BMI and GWG was similar across obstetric groups, their impact on studied outcomes was attenuated among women with a previous PTB or early pregnancy loss. The contribution of BMI and GWG to PTB and SGA exceeded that of prenatal smoking. This is the first study to report population-level contributions of maternal weight to PTB, SGA and LGA in Canada.

Our study adds to existing evidence on the association between maternal weight and adverse neonatal outcomes, by estimating the PAFs of PTB, SGA and LGA that could potentially be prevented if all women began their pregnancy with a normal BMI and had a GWG that was within the recommended range. As the PAF is a factor of both the risk associated with a characteristic and the prevalence of that characteristic, we observed the largest PAFs in subgroups where significant risk and high prevalence converged. For example, the highest odds of LGA (aOR: 2.01[1.12-3.61]) and PTB (aOR: 2.02[1.13-3.63]) were observed among primigravidas with above recommended GWG and primigravidas also had the highest prevalence of above recommended GWG (61.3%), leading to above recommended GWG contributing to a striking 38.9% of LGA and 33.9% of PTB in these women. A similar pattern was observed among multigravid women with no previous PTB or pregnancy loss. However, the pattern varied among women with a previous PTB or pregnancy loss. Among these women, GWG did not contribute significantly to PTB or LGA although the prevalence of excess GWG was similar to that in other groups. This varied pattern is likely a reflection of differences in the distribution of risk factors across groups. For example, the PTB rate in women with a previous PTB was 13.2%, more than twice the population norm (6.1%), suggesting risk factors for PTB not related to maternal weight. The higher risks associated with excess GWG among primigravidas compared to multigravidas, is likely in part attributable to the fact that on average, primiparous women gained more weight during their pregnancy compared to multiparous women.

The utility of the PAF for informing public health prevention policy is illustrated further by the fact that among all women, even though the risk of having an SGA infant associated with underweight BMI was higher than that associated with below recommended GWG (aOR: 2.04 [1.46-2.52] versus 1.56 [1.20-2.03]); the contribution of underweight BMI to SGA was lower than that of below recommended GWG (5.3% versus 9.2%) because fewer women were underweight than below their recommended GWG (6.0% versus 18.6%). Although PAFs are theoretical, as they are based on a particular risk factor being completely eliminated, our results nevertheless illustrate that maternal weight plays an important role in the incidence of neonatal morbidity at the population level. Studying individual associations of risk alone cannot provide evidence of population-level impacts.

Our findings are difficult to compare to those of other studies due to differences in study methods and underlying characteristics of study populations. For example, Oteng-Ntim et al. attributed 4.2% of PTB in a hospital-based study in the United Kingdom to obese BMI [23]; and Djelantik et al. attributed 6.6% of PTB in Amsterdam to overweight or obese BMI [24]. However, neither of these studies controlled for GWG. We found that after adjusting for GWG, overweight or obese BMI did not contribute significantly to PTB, whereas more than recommended GWG contributed to almost one in five (18.2%) preterm births. A South Carolina study which investigated independent contributions of BMI and GWG found that inadequate GWG contributed to 8.1% of very low birthweight (VLBW, 500-1,499 g) and underweight, overweight and obese pregnancy BMI contributed to 8.3%, 3.5% and 7.0% of VLBW respectively [25]. But it is not possible to compare our results, as they dichotomized GWG as less than adequate and adequate (i.e., grouped women with recommended GWG with women with excess GWG) and looked at the impact on low birthweight (LBW) rather than on the constituents of LBW: PTB and SGA, which are known to have different etiologic determinants [6,26]. The importance of looking at PTB and SGA separately, as well as GWG, is highlighted by our findings that excess GWG contributed significantly to PTB but not SGA infants, while underweight BMI and inadequate GWG contributed to both PTB and SGA, but with a larger contribution to SGA. Recognition of such differences facilitates the development of more appropriate preventive interventions for PTB and SGA. Studies have consistently found that obesity contributes significantly to the occurrence of LGA or macrosomia [23,24,27], with PAFs ranging from 7.4% in a UK hospital population in 2004-2008 [23] to 25.7% in Alabama in 1995-1999 [27]. The PAFs of LGA for overweight and obese BMI in our study fell within this range, 6.5% and 8.9% respectively. The large PAF reported in the Alabama study reflects the high prevalence of obesity (≥200 lbs) in this population (21.2% versus 13.5% in our study).

The objective of this study was not only to estimate the contribution of BMI and GWG to adverse neonatal outcomes, but to also compare this contribution to that of prenatal smoking for PTB and SGA in particular. We found that overall below recommended and above recommended GWG contributed more to PTB than prenatal smoking (4.7%, 18.2% versus 3.2% respectively), while underweight BMI contributed less (2.6%). Similarly, below recommended GWG contributed more to SGA than prenatal smoking (9.2% versus 8.7%), while underweight BMI contributed less (5.3%). This pattern varied across obstetric groups, with prenatal smoking contributing to a higher proportion of PTB and SGA than BMI or GWG, among women with a previous PTB or pregnancy loss. As stated earlier, this variation in pattern likely reflects differences in the distribution of underlying risk factors for PTB and SGA across groups. Our results are similar to that of a 2003-2004 study in Amsterdam which found that the contribution of prepregnancy overweight and obesity (BMI ≥ 25) to PTB exceeded that of prenatal smoking (6.6% versus 5.5%) [24]. This study did not investigate contributions of GWG or underweight BMI.

Recognition of the contribution prenatal smoking makes to adverse pregnancy outcomes has led to considerable efforts to develop smoking cessation interventions during pregnancy [28]. However, the contribution of maternal weight to adverse pregnancy outcomes has not been similarly quantified and less attention has been paid to developing interventions aimed at healthy maternal weight during pregnancy. Our results suggest that due to the much higher prevalence of inadequate GWG (18.6%) and excess GWG (59.4%) compared with prenatal smoking (10.4%), maternal weight in general and GWG in particular contributed significantly more to PTB and SGA than prenatal smoking. Thus, from a public health perspective, the importance of healthy maternal weight for the studied outcomes exceeds that of prenatal smoking. Unfortunately, although there is evidence that counselling from a health care provider regarding GWG is effective in helping women plan to gain the recommended amount of weight [29,30], there is also evidence suggesting that few women receive such counselling [31]. In Canada, as in many other high-income countries, prenatal smoking is decreasing [32], while overweight and obesity are increasing [7]. As this trend continues, the already large PAFs for BMI and GWG are likely to increase while PAFs for prenatal smoking decrease. This was observed by Lu et al. in Alabama between 1980-84 and 1995-99. Although there was no significant change in the risk of LGA associated with obesity (≥200 lbs), the PAF of LGA attributed to obesity increased from 6.5% to 19.1%, as the prevalence of obesity increased from 7.7% to 21.2% [27]. Cnattingius et al. asserted in 2002 that from a public health perspective, maternal overweight and obese BMI was one of the most important modifiable risk factors for pregnancy complications and adverse pregnancy outcomes [9]. Our findings support and go beyond this assertion to also highlight the importance of underweight BMI and inadequate and excess GWG.

Our study has several limitations. Self-reported data on BMI and GWG are highly correlated with measured values, but they tend to underestimate these measures [33,34]. This could have led to an overestimation of the risk associated with overweight or obese BMI and excess GWG, as women reporting these characteristics are more likely to be at the higher end of the BMI and GWG spectrum and therefore at increased risk of adverse outcomes [33]. Underestimation of these measures could, however, also have led to more conservative PAFs, as PAFs take into consideration the prevalence of the characteristic. Due to a lack of data on per trimester weight gain, as per IOM guidelines, we assumed a 2 kg weight gain in the first trimester and linear weight gain in the remainder of the pregnancy, although some studies suggest alternate patterns [35]. It is possible that if there was excess gain above 2 kg in the first trimester, that could be contributing to adverse outcomes, rather than second or third trimester excess weight gain. There is a need for more research on timing of GWG and neonatal outcomes. Data did not include a breakdown of PTB by aetiology, so we were unable to assess whether the contribution of maternal weight varied by PTB subtypes [36]. The importance of a previous PTB as a risk factor for adverse outcomes in a subsequent pregnancy may vary with the degree of PTB (e.g. at 31 weeks vs at 36 weeks); however we were not able to assess this as data did not include the gestational age of previous PTBs. Data also only included smoking status in the third trimester. Additionally, studying outcomes overall and across four obstetric groups required us to make multiple comparisons which is known to increase the chance of significant findings [37]. However, the associations we noted are plausible and we reported precise confidence intervals to support interpretation. Finally, our data excluded multiple births and infant deaths, making our population healthier than the general population; and some residual confounding may remain due to unmeasured factors. For example, we did not have information on gestational diabetes which is a risk factor for LGA. However, we note that other data found gestational diabetes contributed far less to LGA (2-8%) compared to excess GWG (33.3-37.7%) [38].

## Conclusions

Our study provides evidence that maternal weight in general, and GWG in particular, contributes significantly to the occurrence of PTB, SGA and LGA in Canada. The contribution of these modifiable risk factors rivals that of prenatal smoking, and the contributions of high BMI and excess GWG in particular are likely to increase as population rates of overweight and obesity rise. These findings highlight the public health importance of promoting a healthy prepregnancy BMI and appropriate GWG.
